# Commentary: Real-world effectiveness and safety of xanomeline and trospium for treatment-resistant schizophrenia in a state hospital system

**DOI:** 10.3389/fpsyt.2026.1807080

**Published:** 2026-03-09

**Authors:** Maxwell Zachary Price, Richard Louis Price

**Affiliations:** 1 Department of Psychiatry, Hackensack Meridian School of Medicine, Nutley, NJ, United States; 2 Department of Psychiatry, Yale School of Medicine, New Haven, CT, United States

**Keywords:** anticholinergics, drug-drug interaction, schizophrenia, treatment-resistant, xanomeline trospium

## 1 Introduction

We appreciated the article “Real-world effectiveness and safety of xanomeline and trospium for treatment-resistant schizophrenia in a state hospital system” by Vadiei N and Crismon ML (2026) (*Front Psychiatry* 16:1736922. doi: 10.3389/fpsyt.2025.1736922) (1). This retrospective study was especially fascinating because the prescribing practices frequently challenged the clinically significant warnings and precautions found in the FDA prescribing guidelines for xanomeline/trospium chloride (KarXT) (2), generating real-world evidence that further reinforces those guidelines for safety and efficacy. Not surprisingly, combining KarXT with medications that can raise xanomeline levels, along with laxatives, increased procholinergic gastrointestinal adverse effects (e.g. nausea, vomiting, diarrhea, sialorrhea) in this study. Likewise, combining KarXT with anticholinergic medications increased peripheral anticholinergic side effects (e.g. blurry vision, tachycardia, gastroesophageal reflux/dyspepsia, constipation, urinary retention) from the trospium component. Additionally, administering KarXT in conjunction with centrally-acting anticholinergic medications attenuated the procholinergic benefits of xanomeline for treatment of schizophrenia symptoms. Consequently, a thorough analysis of this study provides a valuable opportunity to apply FDA prescribing guidelines, extant data, along with our clinical experience treating over 100 patients with KarXT to date, building upon our previously published case series (3), in developing a more nuanced treatment approach to utilizing KarXT successfully. Tailored KarXT adjustments for potential drug-drug interactions (listed parenthetically in this study below), food administration, titration schedule, and prophylactic or rescue ondansetron are useful strategies that can help meet the individual needs of complex patients with schizophrenia, taking multiple medications at baseline.

## 2 Reviewing potential KarXT drug-drug interactions

Firstly, it is important to consider potential pharmacokinetic interactions affecting plasma exposure, Cmax (maximum concentration) and AUC (area under the curve) of xanomeline and trospium when prescribing KarXT in combination with other medications (4). According to the KarXT package insert, use in CYP2D6 intermittent/poor metabolizers or concomitant use with strong hepatic CYP2D6 inhibitors (2) (e.g. strong-bupropion, terbinafine; moderate-sertraline, haloperidol, hydroxyzine) may increase systemic plasma concentrations of xanomeline, which may increase frequency and/or severity of xanomeline-related adverse reactions. For example, based on a population pharmacokinetic analysis, median Cmax and median AUC of xanomeline increased approximately 28% and 15%, respectively, in intermediate metabolizers (2). Further, xanomeline transiently inhibits both CYP3A4 and P-glycoprotein locally in the intestine (first-pass), which may increase plasma concentrations of oral sensitive substrates (2) (e.g. CYP3A4 substrates-clonazepam, metoprolol, haloperidol, hydroxyzine, aripiprazole, risperidone, clozapine, benztropine, quetiapine, terbinafine, lurasidone, suvorexant, trazodone, amlodipine, melatonin, tamsulosin, loratadine, chlorpromazine, buspirone, mirtazapine; P-glycoprotein substrates-aripiprazole, risperidone, paliperidone, quetiapine, valsartan, amlodipine, famotidine, tamsulosin, loratidine, lorazepam, sitagliptin), increasing the frequency and/or severity of adverse reactions from those substrates. Drugs eliminated by active tubular secretion (2) (e.g. amantadine, metformin [decreases trospium], clozapine, atropine, famotidine, glycopyrrolate, nicotine, sitagliptin) may increase plasma concentrations of trospium, and/or the concomitantly used drug, which may increase the frequency and/or severity of adverse reactions.

Secondly, it is also important to consider potential pharmacodynamic interactions of co-prescribed anticholinergic medications affecting additive anticholinergic burden with trospium and receptor-level competition affecting procholinergic benefits with xanomeline. Concomitant use of KarXT with other anticholinergics (2) (e.g. glycopyrrolate) may increase the frequency and/or severity of peripheral anticholinergic side effects. Moreover, the mechanism of action of KarXT is thought to be related to its central agonist activity at M1 and M4 muscarinic acetylcholine receptors, with comparable affinity at M1-M5 (Ki=10, 12,17, 7, and 22 nM, respectively) (2), such that combining KarXT with central M1 and M4 antagonists in the nM range (e.g. olanzapine, clozapine, quetiapine, atropine, chlorpromazine, benztropine, diphenhydramine) can attenuate efficacy. However, individual results can vary as the Ki values for xanomeline may be lower than several anticholinergic antipsychotics used successfully with KarXT in treatment-resistant schizophrenia (TRS) (5), and certainly for concomitant non-anticholinergic antipsychotics in TRS (6). Ki values need to be interpreted in context of *in vivo* receptor occupancy and activity, which also depends on free plasma concentration, blood-brain barrier penetration, and dynamic pharmacokinetic factors.

## 3 Food administration and titration schedule

KarXT should be administered twice daily at least one hour before meals and at least two hours after meals because trospium’s AUC absorption can be reduced 85%-90% with a high fat meal, and xanomeline’s AUC can be increased 30% compared to fasted state (2). This imbalance may transiently amplify procholinergic effects during early titration by relatively reducing peripheral antagonism, while modestly increasing central and peripheral agonist exposure from unopposed xanomeline. Nevertheless, the authors note that KarXT was administered without regard to this food warning, which explains the high rate of xanomeline-related procholinergic side effects, limiting tolerability and often curtailing an adequate trial to capture the full benefits of KarXT (1). This warning is especially important during the first 4 weeks of dose titration, when procholinergic side effects can predominate for a couple weeks until tolerability develops, especially when moving from the 50 mg xanomeline/20 mg trospium dose to the 100 mg xanomeline/20 mg trospium dose, where the xanomeline dose doubles, yet the trospium dose remains the same. This is further mitigated by titrating up to the highest dose of 125 mg xanomeline/30 mg trospium. Following the first 4 weeks, taking KarXT with food can help mitigate anticholinergic side effects of trospium by limiting its absorption (7, 8). This is a useful strategy in patients who have anticholinergic side effects but require strongly anticholinergic antipsychotics for stability while titrating KarXT to full effect, prior to gradually tapering these meds. Conversely, stable patients have been able to successfully taper off previous antipsychotics over 2 weeks prior to initiating KarXT (8).

## 4 The role of ondansetron

Given that the procholinergic side effects of xanomeline tend to predominate during KarXT initiation and the initial titration, using prophylactic or as needed (PRN) ondansetron has been largely effective in real-world TRS practice. In a retrospective analysis of 90 patients initiating KarXT while taking antipsychotics, prophylactic (21%) or PRN (44%) ondansetron yielded improvement in nausea or vomiting (71%). This includes those transitioning from clozapine (11%), olanzapine (24%), or other antipsychotics in helping patients stay on treatment until these side effects abate, without adding further anticholinergic burden from other antiemetics. Adverse effects from the addition of ondansetron, such as QT-prolongation, were not observed (9). Unfortunately, ondansetron was utilized in only one patient in this study (1, Supplement).

## 5 Discussion

While we appreciate the courage to add KarXT to complicated medication regimens in patients suffering with TRS, concluding that KarXT would necessarily be intolerable and ineffective in this population may be more reflective of implementation factors (**Table 1**). The authors acknowledge that the retrospective design may limit causal inference and that illness severity, institutional prescribing constraints, and baseline comorbidity burden may have also influenced outcomes (1). Nonetheless, the majority of patients (80%) were treated off label for a diagnosis of schizoaffective disorder rather than schizophrenia, potentially necessitating treatments other than KarXT. Notwithstanding that ondansetron was rarely provided, all reports of nausea, vomiting, diarrhea could have been explained by taking KarXT with food, which is the highest priority and most effective means to mitigate these side effects. Careful attention needs to be paid to drug-drug interactions that can either raise xanomeline exposure and side effects (CYP2D6 inhibitors), raise levels of other medications and side effects (intestinal CYP3A4 and P-glucoprotein substrates), compete for active tubular secretion, or add to anticholinergic side effects and may attenuate xanomeline’s first-in-class putative mechanism of action.

**Table 1:** Clinical case considerations from supplement (1).

Case	Adverse effects reported	Mitigation efficacy strategies
1	N/A	Increase X/T from 100/20 to 125/30. After 4 weeks, slow taper off olanzapine to unmask full benefits of X/T for positive symptoms and for negative/cognitive symptoms that olanzapine can exacerbate.
2	N/V/D	Take X/T without food. Ondansetron 8 mg BID until N/V/D resolved. Consider lowering sertraline, which can raise xanomeline exposure. Slow taper off olanzapine as above. Taper amantadine due to increased exposure via active tubular secretion competition from trospium.
3	Emesis	Take X/T without food. Ondansetron 8 mg BID as above. Use alternative sleep aid/anxiolytic as hydroxyzine can increase xanomeline exposure. Slow taper off olanzapine as above.
4	Diarrhea	Take X/T without food. Taper off bupropion, which can raise xanomeline exposure. Change metformin, which can decrease trospium levels and cause diarrhea. Discontinue docusate, Miralax, and lubiprostone, which can cause nausea, diarrhea, abdominal pain, and bloating.
5	Only nauseated at night, fine during the day	Take X/T without food-1 hour before breakfast, 2 hours after finishing lunch and 1 hour before dinner, as people tend to eat at night and forget when they finished eating before bed. Slow taper off clozapine to unmask full benefits of X/T.
6	Uncontrolled vomiting	Take X/T without food. Change terbinafine, which can increase xanomeline exposure. Ondansetron 8 mg BID as above. Slow taper off quetiapine to unmask full benefits of X/T. Taper amantadine due to increased exposure via active tubular secretion competition from trospium.
7	Nausea	Take X/T without food. Ondansetron 8 mg BID as above. Slow taper off olanzapine as above.
8	Seizure, incontinent of feces, dystonic reaction, vomiting	Reduce risperdal and haloperidol, can reduce seizure threshold and cause dystonia, both 3A4 substrates increased by xanomeline. Discontinue lubiprostone, Miralax, docusate-senna in context of fecal incontinence Take X/T without food. Ondansetron 8 mg BID as above. Increase X/T to 125/30 for additional trospium.
9	N/A	Without food, increase X/T from 50/20 to 100/20 for 1 week as tolerated, to maximum effective dose of 125/30.
10	Constipation	Taper off atropine and famotidine, which exacerbate constipation. Consider X/T with food to reduce trospium absorption with olanzapine. Titrate X/T to 125/30, then slow taper off olanzapine as above to further reduce constipation
11	Vomiting and constipation	Discontinue loratadine, 3A4 and P-glycoprotein substrate, higher levels causing abdominal pain, nausea, increasing trospium exposure. Ondansetron 8 mg BID as above. Take with food if constipation predominates; take without food if vomiting. Titrate to 125/30 as tolerated.
12	Sleepy	Taper clonazepam, melatonin, 3A4 substrates, higher exposure causing sedation. Titrate X/T to 125/30 as tolerated.
13	Itchy red patches, flaking and rash 6 days after XT initiation, on 100/20 dose	Discontinue X/T due to potential serious allergic reaction to trospium. Glyopyrrolate may raise trospium exposure due to active tubular secretion competition.
14	Nausea on 100/20 with ondansetron 4 mg BID together before meals	Verify X/T taken 1 hour before meals and at least 2 hours after finishing the previous meal. Increase Ondansetron 8 mg BID. Increase X/T to 125/30 for additional trospium. Slow taper off benztropine and olanzapine as above.
15	Sialorrhea	Take X/T without food. Taper chlorpromazine, raises xanomeline exposure (2D6), levels raised by xanomeline (3A4, P-glycoprotein substrate). Then titrate to 125/30 for additional trospium.
16	Dizziness, blurring vision on 50/20. Diarrhea on 100/20	Take X/T without food. Taper bupropion increases xanomeline exposure. Increase X/T to 125/30 for additional trospium. Taper off glycopyrrolate to reduce anticholinergic dizziness, blurred vision. Taper off benztropine to unmask full benefits of X/T.
17	Urinating on himself, incontinence, appearing overmedicated/oversedated on 100/20	Take X/T without food. Titrate to 125/30; additional trospium helps urinary incontinence To alleviate sedation, taper chlorpromazine, raises xanomeline exposure (2D6), levels raised by xanomeline (3A4, P-glycoprotein substrate), taper clonazepam (3A4 substrate), taper diphenhydramine.
18	N/A	Increase X/T to 125/30 without food. Taper off benztropine and diphenhydramine to unmask full benefits of X/T.
19	Hyperreligious, hypersexual, irrational, grandiose, and delusional	Mania with psychosis may require different treatment than X/T, approved for schizophrenia, not schizoaffective. Increase X/T to 125/30, but diphenhydramine, olanzapine attenuate effects. Higher olanzapine may be indicated
20	No side effects, no benefit on 125/30	Taper off amantadine (raises dopamine) due to increased exposure via active tubular secretion competition from trospium.

X/T, xanomeline and trospium chloride; N/V/D, nausea/vomiting/diarrhea; BID, twice daily; N/V, nausea/vomiting.
